# Supplementary figures and images for: In vitro evidence against productive SARS-CoV-2 infection of human testicular cells: Bystander effects of infection mediate testicular injury
Source: PLoS Pathog. 2023 May 18;19(5):e1011409. doi: 10.1371/journal.ppat.1011409 (PMC10231791; doi:10.1371/journal.ppat.1011409)

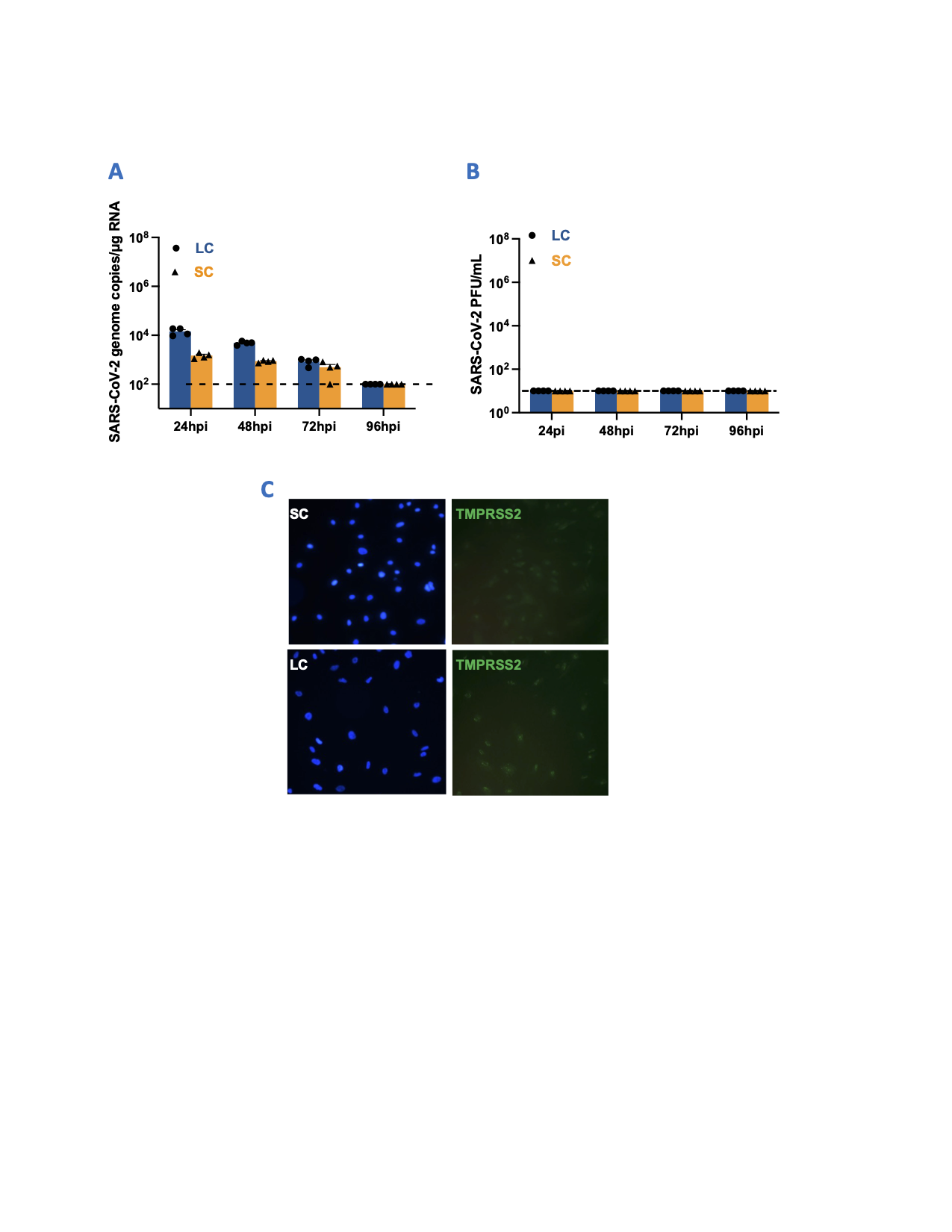

Supplement: S1 Fig — Primary SC, LC, STC, and HTO were infected with SARS-CoV-2 at MOI 10 and (A) intracellular virus levels were determined using qRT-PCR and (B) virus titers in the supernatant were measured using plaque assay (C) Representative TMPRSS2 (green) and DAPI (blue) staining of primary Sertoli (top) and Leydig (bottom) cells. (TIFF) [file ppat.1011409.s001.tiff]

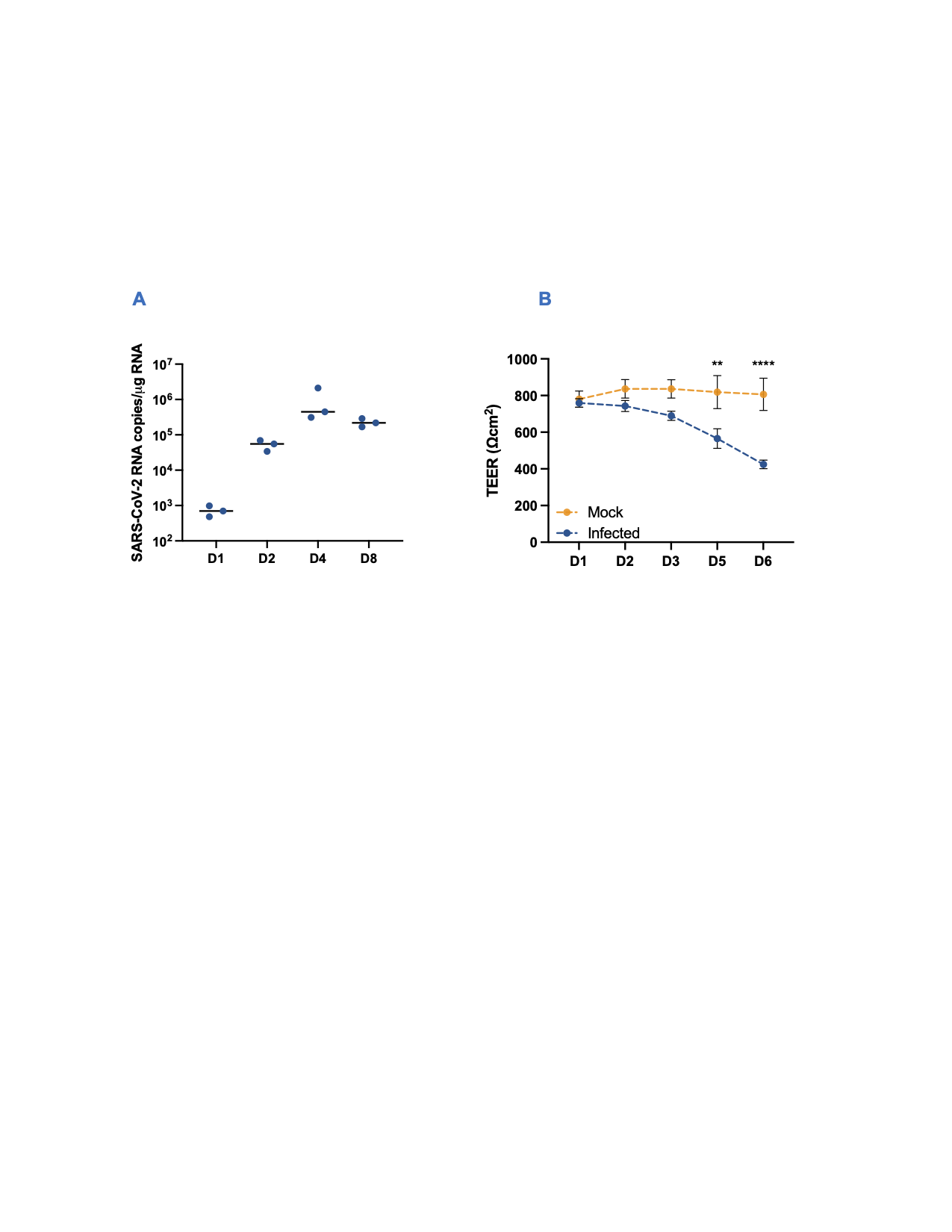

Supplement: S2 Fig — (A) SARS-CoV-2 RNA was measured in HAE cells at days (D) 1, 2, 4 and 8 post-infection using qRT-PCR (B) The transepithelial electrical resistance (TEER) was used to measure the integrity of the air-liquid barrier of HAE inserts at different days post-infection (MOI 1), and expressed in Ohm*cm2 (Ωcm2) **p<0.01; ****p<0.0001 compared to mock. (TIFF) [file ppat.1011409.s002.tiff]

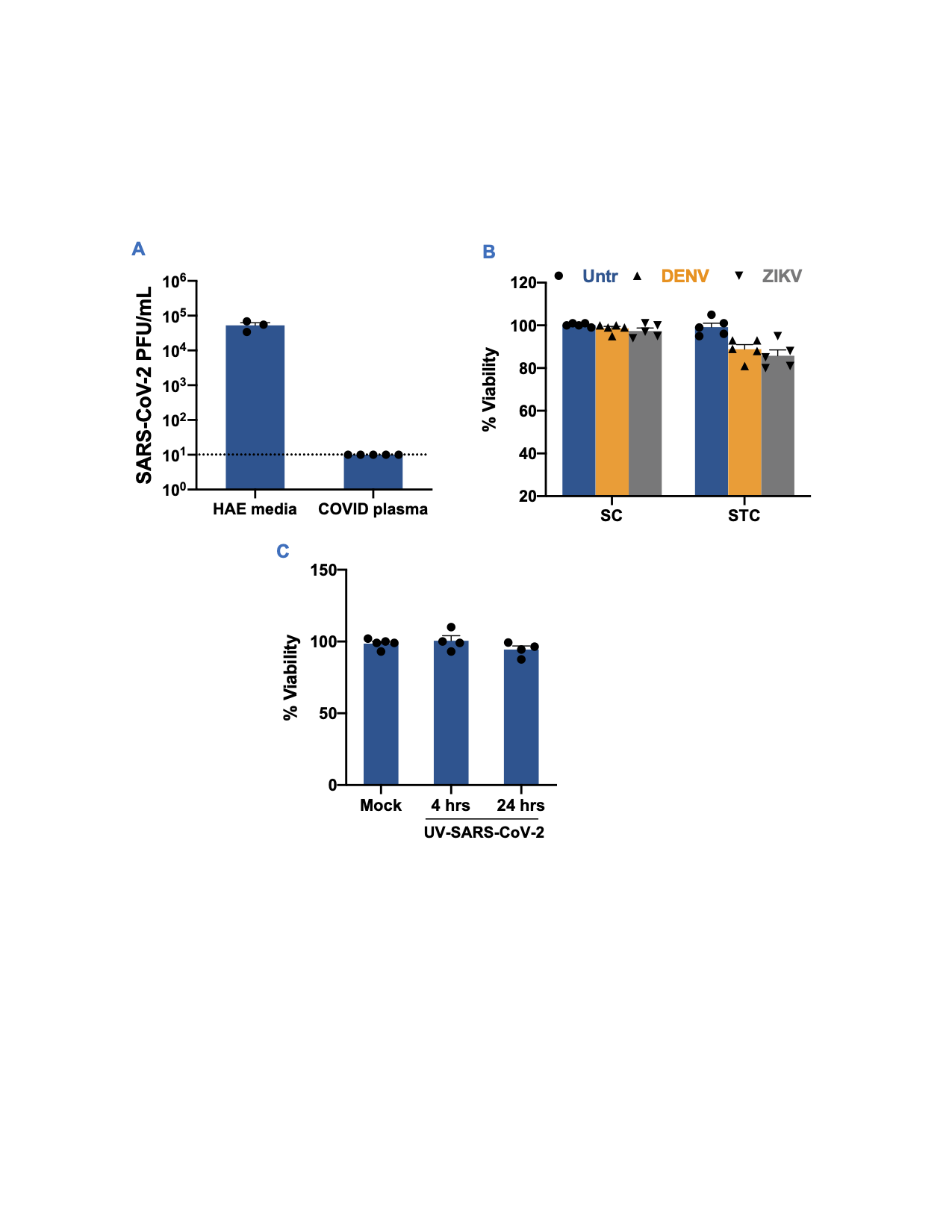

Supplement: S3 Fig — (A) Progeny virus titers in the basal media of HAE cells (2 dpi as control) and serum of COVID-19 patients demonstrating no virus in the patient sera (B) STC were exposed to UV-inactivated supernatant from dengue virus (DENV)- infected HUVEC and Zika virus (ZIKV) infected SC and viability was calculated after 24hrs. (C) SC were exposed to UV-inactivated virus stock (1.17x10^7^ PFU/mL) and viability was calculated at 4hrs and 24hrs. (TIFF) [file ppat.1011409.s003.tiff]

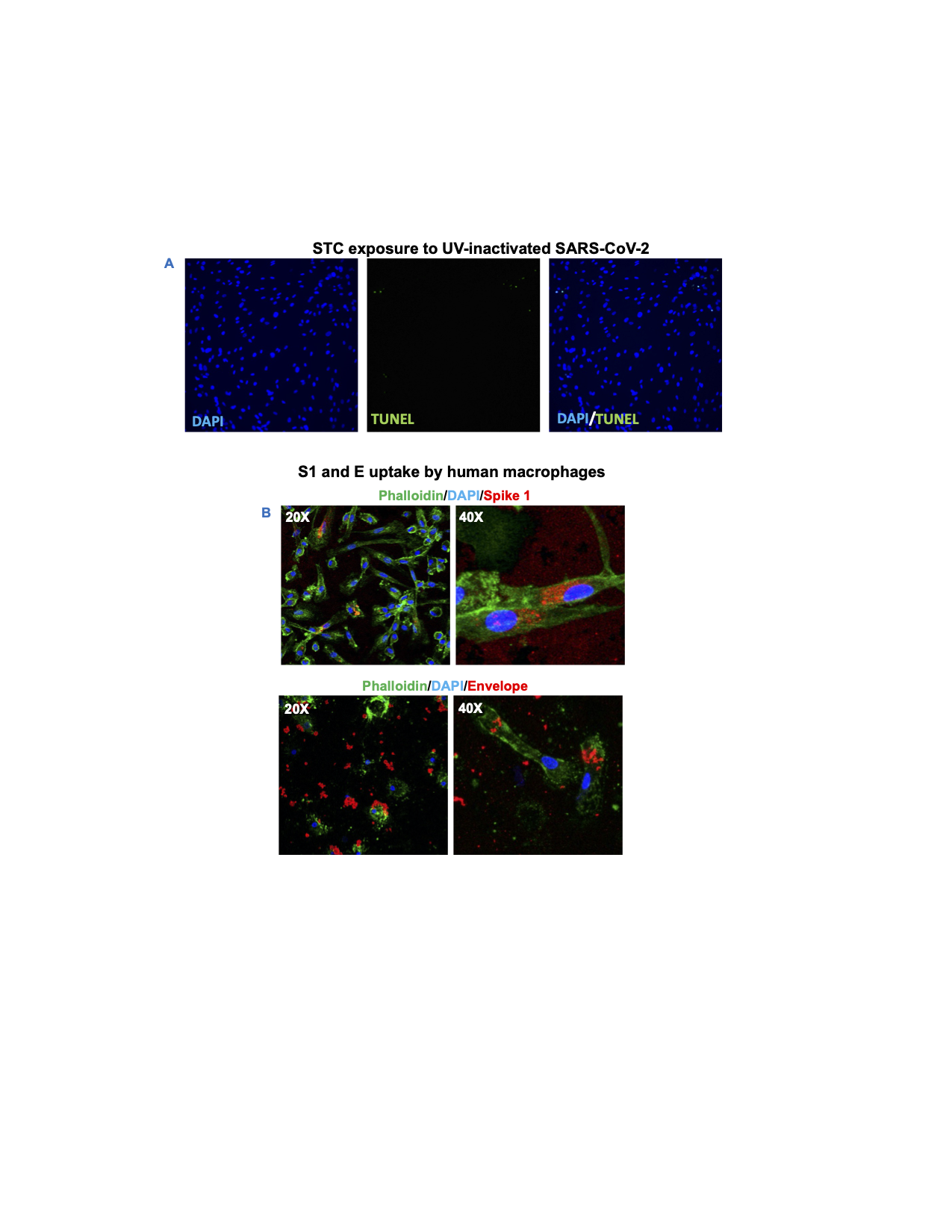

Supplement: S4 Fig — (A) Representative TUNEL staining in STC exposed to UV-inactivated virus stock (107 PFU/mL) for 24 hrs. The green fluorescence depicts TUNEL+ cells and shows no cell death (B) Human macrophages were exposed to red fluorophore-conjugated E and S1 proteins (1ng) and the uptake was evaluated after 24 hrs by detecting intracellular virus antigens (red) following staining with DAPI (blue) and Phalloidin (green), a marker for actin filaments. High-power magnification pictures depict the uptake of S1 and E by cells while the loss of cytoskeleton was evident only in E-treated cells. (TIFF) [file ppat.1011409.s004.tiff]
