# Supplementary material for: In vitro evidence against productive SARS-CoV-2 infection of human testicular cells: Bystander effects of infection mediate testicular injury
Source: PLoS Pathog. 2023 May 18;19(5):e1011409. doi: 10.1371/journal.ppat.1011409 (PMC10231791; doi:10.1371/journal.ppat.1011409)
Supplement: Attachment — Submitted filename: Reviewer Comments -4-28-23-FINAL.pdf [file ppat.1011409.s005.pdf]

Reviewer's Responses to Questions

We thank the reviewers for their constructive comments. In general, all the reviewers felt that this manuscript is 'well done', 'the data supports the overall conclusions', this 'research provides evidence to explain the clinical manifestation of testicular symptoms associated with severe COVID-19', and 'brings insights into the potential mechanism'. However, they all felt that further experiments are required to strengthen the work presented. We have tried to address all concerns by either conducting new experiments or giving an additional explanation as described below.

New experiments include:

i. SARS-CoV-2 RNA in cells treated with exogenous serine protease at early time point (12hrs). New data in Fig 1D
ii. SARS-CoV-2 assessment in the COVID-19 plasma (Fig. S3A)
iii. Cell viability in STC treated with UV-inactivated virus stock to determine if exposure to virus proteins associated with virions can induce cytotoxicity (Fig. S3C)
iv. Exposure of STC to conditioned media from SC infected with Zika virus to address the concern that is this specific to SARS-CoV-2 (Fig. S3B).
v. Cytotoxicity experiments in E-treated cells in the presence of neutralizing antibodies against TLR1 and TLR6 to further confirm that it is via TLR2 (Fig. 5H)
vi. Uptake of S1 and E in human macrophages to depict that the internalization of S1 in these cells is higher than SC to justify the disparity between this and other studies (Fig. S4B)
vii. Measurement of S1, N, and E levels in the mouse serum at different time points after infection (Fig. 7E)
viii. Added more data points in the mouse study (IL-6 and TNF-α RT-PCR analysis in the Figure 7D)
ix. SARS-CoV-2 RNA and virus titers measured in the brain (Fig. 7B-C)

Reviewer #1:

Major comments:

Comment 1: The description "supernatant from HAE" is easy to mistake for the apical wash, especially when the authors did not mention it is from the basal side in figure legends. Authors may change it to basal media or something else.

Response: Thanks, yes, the suggested change has been made and it is clarified that supernatant from HAE is the basal media.

Comment 2: My major concern is the difference between basal media from infected HAE and plasma from COVID-19 patients. Except for the biological differences, the basal media, in this case, contain a large number of viral particles. Did authors find virus in patients' plasma?

Response: As suggested by the reviewer, we measured virus mRNA in the plasma samples. As expected, SARS-CoV-2 was not detected in the plasma which supports other similar studies. We have made it clear in the text.

Comment 3: Even after UV inactivation, the viral particles may influence cells…. ….. Did authors try directly applying UV-inactivated viral particles to STC cells? What is the protein level of viral antigens (S, E, N) in plasma from COVID-19 patients or basal media from infected HAE? Could the concentration of E protein be over 1ng/ul? In patients, the antigens are from secreted proteins or viral particles?

Response:

i. Thanks for the suggestion. This experiment was performed, and STC were exposed to UV-inactivated virus stock (10^7 PFU/mL) for 4 and 24 hrs and we did not observe any cytopathic effects (data included in Supplemental figure S3C).
ii. Regarding the levels of virus particles, we measured E (and S1) protein in HAE basal media and COVID plasma. Both E or Spike were not detectable in HAE basal media, and we speculate that the concentration of these proteins secreted from HAE cells is too low and diluted in the media and is therefore below the detection limit of the commercially available kits. Therefore, we speculate that in HAE basal media-treated cells, cell death is mainly due to inflammatory mediators.
iii. We did find detectable S1 in the COVID-19 plasma samples tested. We have another project ongoing in which we plan to measure SARS-CoV-2 antigens (S1, N and E) in the plasma of a large cohort of COVID-19 samples and their correlation with disease severity, and this data will be part of another manuscript. Therefore, we will not be able to include this data in this paper. However, we measured virus protein levels (S1, N, and E) in the serum from K18 mice at days 3, 5, and 8 after infection using commercially available ELISA kits. This is the first study to report virus antigen levels in mouse sera that at least for the Spike protein follows a similar trend as seen in humans (shown in Fig 7E). We have clarified the discussion a little bit to reflect this and suggest that unlike HAE media-exposed STC, the cell death in COVID-19 plasma-treated cells is most likely due to a combination of virus proteins and inflammatory mediators in the plasma.
iv. The dose of different proteins used in our study is based on other similar in vitro studies (1). Spike levels in the plasma of COVID patients are shown to be in the range of 10-1000pg/mL plasma (2), while the in vitro studies including our study have used proteins in the range of 0.5-4 μg/mL (0.5-4 ng/μL).

Comments 4: Fig1D, except for plaque assay, did authors also check the viral RNA level at an earlier time point (eg. 12hpi)? If the gRNA copy number is higher in +SerP group, it may hint the viral entered cells better in presence of exogenous serine protease but did not replicate because of the lack of some host factors.

Response: As suggested, this experiment was done, and the viral RNA copy number is now included in the new Fig 1D.

Minor comments:

Comments 5: Fig 1B, I believe at MOI=1, all Vero cells must die at 96hpi. Please interpret in figure the dotted line represents the limit of detection (LOD).

Response: Yes, almost 80% cells are dead by 96 hpi. However, since the media is not changed every day, the virus in the supernatant at 96 hi is the total remnant virus secreted at 48 or 72 hpi in the Vero cell media. We have also explained the LOD in the legend.

Comments 6: In SARS-CoV-2 infected K18-hACE2 mice, authors described testicular edema, germ cell disorganization, and congested tubules were observed in some areas. It is not sure how often this is observed. If they can provide a disease score system to quantify that, it will help readers to have a big picture.
Response: Thanks for the suggestion. We have now included quantitative data from at least 3-6 mice per time point. The scoring strategy is based on the study previously published (3) by our collaborator. The data is based on 50 randomly selected tubules from each section and tubules were classified as abnormal if they showed (i) cells in lumen; (ii) lack of clear lumen (iii) separation from the basement membrane and (iv) apoptotic cells. The data is now included in the new Fig 7G.

Comments 7: The Fig4C, COVID-19 Plasma pictures are not very convincing for colocalization.
Response: We have replaced it with a new figure from another coverslip.

Reviewer #2:

Major comments:
Comments 1: Previous report suggest S1 mediated TLR2 activation is sufficient to induce inflammatory response (PMID- 34866574). As in this case S protein failed to induce inflammatory response via TLR2, the disparity in data should be explained. The repeat experiments with concurrent exposure to S1, S2, M, N, and E proteins would provide a more comprehensive picture of the inflammation in these cells.
Response: In the referenced study PMID- 34866574 by Khan et al. (1), THP1, monocytic cell line, and mouse BMBDs were mainly used to depict the inflammatory response to S1. The treatment dose of S1 in our study is comparable to other similar studies, however, there can be different reasons to explain this disparity-

i. Monocytic cell lines being phagocytic in nature may respond differently with respect to the uptake of Spike protein compared to the primary testicular cells used in our study which may result in a difference in downstream response.
ii. The data on S1 response on different cell types is conflicting. Another study by Zheng et al. showed that exposure of mouse BMDMs to S1 did not induce an inflammatory response (4). In vivo data from the same group indicated that only Envelope protein and not Spike protein induced lung inflammation. Our data agrees more with this study.
iii. Other cell types tested by Khan et al. were also cell lines like Calu and A549 cells. Therefore, another reason for the disparity between different groups may also be attributed to the use of cell lines vs primary cells used by us.
iv. To further clarify the reviewer's concern, we exposed human macrophages to fluorophore-conjugated spike and envelope proteins as shown in Supplement Fig. S4B. Our data show that human macrophages internalized S1 but without a significant effect on the cytoskeleton although the levels of S1 uptake was much lower than internalized E protein in these cells.

We have further clarified the discussion to explain the disparity between different studies.

Comments 2: TLR2 is known to form a heterodimer with either TLR1 or TLR6 on the cell surface that promotes ligand binding and signal propagation. The basic mechanism of inflammatory response due to E-protein interaction with TLR2 should be investigated.
Response: Thanks for the suggestion, As recommended, new experiments were conducted using the neutralizing antibodies against TLR1 and 6. The data is included in the new Fig 5H and shows that the response is dependent on TLR2 only.

Comments 3: As testis in in-vivo system are not being infected by SARS-CoV-2, how does the E- protein interacts with the TLR2 on the testicular cells? Is there any evidence of interaction between TLR2 and E protein in the in vivo system? The biological relevance of this study is not clear.
Response: Two in vivo studies provide evidence that E protein can induce lung pathology and one of them shows it is TLR2 dependent (4, 5). These studies are described in the discussion to further clarify their relevance. The biological relevance of this study is to show that despite the absence of infectious SARS-CoV-2 in the testis, inflammation and morphological alterations are observed. Although our new mouse data demonstrate the presence of E protein in the serum, E protein levels are not yet reported in COVID-19 patients. Therefore, we have further clarified the discussion. We now attribute systemic inflammation as the main driver of testicular injury and virus antigens as another contributory factor depending on their levels to this damage.

Comments 4: Did author investigate the expression profile of TLR2 in hACE2-K18 mice? Did Authors investigate testis pathology after TLR2 inhibitor administration in these mice? Infection of TLR2 KO/ TLR2 conditional KO mice with mouse adapted virus may provide better insight about the role of TLR2 in testicular inflammation.
Response: We agree with the reviewer's thoughts. The expression of TLR2 in the mouse testis and its role in testicular inflammation in the context of viral infection is well established in viruses like mumps virus (6, 7). Similar studies as suggested by the reviewer have been conducted recently by Kanneganti Lab (4). In this study, the authors showed that TLR2 sensed the SARS-CoV-2 envelope protein as its ligand. In addition, blocking TLR2 signaling in vivo provided protection against the lung pathogenesis of SARS-CoV-2 infection. However, this study did not assess pathology in other tissues including the testis. They also showed that mouse coronavirus MHV infection in TLR2 KO BMDMs leads to a decreased inflammatory response. Interestingly, they also showed that only E but not Spike protein leads to the induction of TNF-α, IL-6, and IL-1β. This data supports our in vitro data. However, conducting new experiments using mouse-adapted strain is beyond the scope of this study as we don't have access to mouse-adapted strain. However, we plan to further characterize the SARS-CoV-2-associated testicular injury in vivo and will consider studying the impact of E on testicular injury in the future. We have included these references in the text.

Comments 5: Figure 7D. TNF and IL-6 shows increased expression at 5dpi. Are these statistically significant?
Response: We have now conducted TNF-α and IL-6 gene expression analysis in additional mice (now total 5-6 mice for each time point) and the new data (Fig 7D)

shows that the difference is statistically significant compared to mock-infected testis. We have included the p-value in the figure and legends.

Comments 6: Deleterious effect of inflammation has been well established. In this work, authors have showed deleterious effect of inflammatory cytokines on testicular cells. Why do authors think that the SARS-CoV-2 E protein is major source of damage in testicular cells as compared to systemic inflammation induced due to the direct infection in lungs?
Response: We believe that testicular damage is due to a combination of both -systemic inflammation and virus proteins and that systemic inflammation plays a dominant role in the injury. We speculate that the levels of the envelope protein and robustness of the cytokine storm may vary from patient to patient thus affecting the upstream trigger of the testicular injury. Our ongoing study which will be communicated separately is focused on measuring the levels of all three virus antigens and their correlation with systemic storm and disease severity.

Our in vivo data shows levels of all three virus proteins including E protein vary greatly in the plasma at day 5 which follows a similar trend seen in humans for S1 and N. And yes, it is true that inflammation-associated tissue damage is well-known in different infectious and non-infectious diseases like sepsis. Similarly, virus antigens like NS1 and glycoprotein of dengue and Ebola viruses are associated with enhancing inflammatory response and cell injury. Based on these collective studies and our data, we speculate that both systemic inflammation and circulating virus proteins can independently or collectively cause testicular damage depending on their levels. However, the relative contribution of inflammatory cytokines vs Envelope protein cannot be determined in this study. The discussion is modified to make it clearer.

Minor comments:
Comments 7: Line 155-156. Reference should be added.
Figure 2D Statistical analysis should be shown.
SARS-CoV-2 E-protein generation and purification should be mentioned in Method section
Response: Suggested changes have been made in the revised text. Thanks

Reviewer #3:

Major comments:
Comments 1: Comparison with influenza infection, as an unrelated respiratory virus that also causes systemic inflammation, or similar model is important for the interpretation of the results presented in this manuscript.
Response: Yes, we agree that this comparison is important to understand why SARS-associated inflammation causes more multi-organ injury than influenza. Severe influenza is also known to cause complications including myocarditis, encephalitis, or myositis. However, there are no reports of testicular injury or orchitis symptoms in influenza patients during the acute stage of the disease or after recovery.

Systemic cytokine storm is well described in influenza-infected patients and a recent study compared the cytokine profile with COVID-19 patients (8). The data shows that while IL-2 is exclusively produced by influenza, cytokines like IL-6, TNF-α, and IL-1β are produced by both viruses, though levels are higher in COVID-19 patients. While IL-4, IL-9, CCL5, CCL8, GM-CSF, and PDGF are exclusively induced by SARS-CoV-2. Also, TWEAK, an amplifier of IL-6 is also upregulated by SARS-CoV-2. Therefore, all these differences can be attributed to more severe multi-organ injury including testicular damage and long-term sequelae observed in COVID-19 patients. We have modified the discussion to reflect this aspect of interpretation.

Comments 2: Questions about the physiologic relevance and biological plausibility of E protein stimulating changes in testicular cells must also be answered.
Finally, it is well known that SARS-CoV-2 infection stimulates cytokine and chemokine production including IL-6, TNF-α and IL1-β and that systemic inflammation can lead to vascular permeability and deleterious effects on a wide variety of cell types and systems which lessens the novelty of this work. A more detailed mechanism would raise the impact of this manuscript.
Response: As described in some of the responses to reviewers 1 and 2, there are few reports on Spike and N protein levels in the plasma of COVID-19 patients, but no study has looked into E protein levels. However, several studies have assessed the cytotoxic role of E protein both in vitro and in vivo and E concentration used in our study is based on these in vitro studies (1, 9). However, our new mouse data shows levels of all three virus proteins including the E protein vary greatly in the plasma and at this point, the correlation between antigen levels and testicular injury cannot be delineated. Therefore, we have now clarified in our discussion that systemic inflammation plays a dominant role in the injury, while E protein can further contribute to the injury (Rev# 2, comment 6).

Regarding novelty, the new insights this study provides are that (i) testicular injury is not due to direct virus infection as there are conflicting reports on this issue (ii) direct evidence of the cytotoxic effects of systemic inflammation and virus proteins. New data provides new insights into the mechanism of action of E protein on SC (iii) we established the K18 mice model to study the bystander effect of infection on the testicular injury for the first time. Our data shows testicular injury mimics what is observed in the human testis. This model will allow us to further characterize injury post-recovery as compromised male reproductive health is now included as one of the long-COVID symptoms. (iv) Lastly, new data for the first time measured the levels of SARS antigens in mouse sera.

However, we agree that more detailed insights into the mechanisms of injury are needed. Ongoing studies include RNA-seq of mouse testis at different stages of disease (early, acute, and 3-4 weeks after recovery) and that data along with histopathology analysis at same time points will be communicated separately after proper validation.

Comments 3: The K18 mouse model description is overstated and fails to mention that the severe disease that mice die of is encephalitis and not respiratory. As such, this

model is limited in its use for pathogenesis studies. It is important that the authors examine RNA and virus load in the brain in these experiments.
Response: We agree. It's true that K18 mice exhibit high virus infection of the brain in addition to the lungs (10, 11). However, it is considered that the systemic cytokine storm is due to virus infection and infiltration of immune cells in the lung. As suggested, we have included brain RNA and virus titers in the new Fig 7B and C. Our data follows the same trend as shown in the studies referenced above. We also believe that virus infection in the brain does not change the suitability of this model to study bystander effects on the testis. Since K18 mouse testis does not show virus RNA or infectious virions, and levels of key systemic cytokines are similar to COVID-19 patients, it serves to test our hypothesis. We have further modified the discussion to further clarify brain infection in this model.

Comments 4: The pathology in Figure 7 is some of the most important data for the conclusions of this manuscript. Is there a scoring scheme/quantitation that can be applied to these samples?
Response: Thanks for the suggestion. As described in our response to reviewer # 1, comment 6, our collaborators have expertise in characterizing testicular abnormalities as shown in the previously published manuscript (3). we have now included quantitative data from 3-6 mice per time point. The data is based on 50 randomly selected tubules from at least 3 sections taken throughout the length the mouse testis of each male and tubules were classified as abnormal if they showed (i) cells in lumen; (ii) lack of clear lumen (iii) separation from the basement membrane (iv) apoptotic cells. The data is now included in the new Fig 7.

Comments 5: Is this testicular cell injury phenotype unique to SARS-CoV-2 infection? Would media from influenza (or other broadly inflammatory virus) infected cultures have the same effect on testicular cell viability?
Response: Viruses like Zika, Ebola and Marburg virus can directly infect testicular cells and trigger systemic inflammation, therefore the bystander effect of cytokines on testis is not clear. Influenza virus triggers systemic cytokines similar to SARS-CoV-2, however, as shown by Pacheco-Hernández et. al (8), inflammatory cytokines levels are much higher in COVID-19 patients, that may explain why influenza patients do not report orchitis symptoms and altered fertility markers. However, similar testicular injury was reported in the patients infected with SARS coronavirus, similar of SARS-CoV-2 (12) suggesting that both these coronaviruses can induce gross alterations in the testis. Since we do not have access to the influenza virus, we tested the cell viability of STC cells exposed to conditioned media from ZIKV-infected STC (a virus that infects testicular cells) and Dengue-infected HUVEC cells (a cell type where this virus is shown to induce proinflammatory cytokines). Data shows that media from both these cells reduced the viability by only 10-15% compared to respective mock controls. This was much lower than what is seen in the presence of HAE basal media thus suggesting that both coronaviruses SARS-CoV and SARS-CoV-2 can induce testicular pathology that is not a feature of influenza virus.

Minor comments

Comments 6: If exposure to E protein alone is enough to induce cytokine secretion (Fig 5D-G), why was exposure to virions not able to induce the same response (Fig 1E)? Is there any evidence of circulating E protein in the blood of COVID-19 patients? How would a transmembrane protein be stable in circulation?
Response: As explained earlier, there is no evidence of circulating E protein in COVID-19 patients. However, our ongoing study plans to measure this antigen in a local cohort of COVID1-9 patients. It is suggested that infected and dying lung cells during cell lysis may secrete these antigens as part of immature virions or free proteins. But we do have evidence now that E is secreted in the serum of infected K18 mice though the levels vary greatly from mouse to mouse. This data is now included in the new Fig 7E.

Comments 7: Figure 2- Similar data has been previously published and this would be more appropriate as a supplemental figure
Response: As suggested, we have moved two figures to the supplemental figure and have included a new figure (Fig. 2C) of the Luminex data of the comparison of multiple cytokines in apical and basal media at both time points. To our knowledge, this in-depth data is not reported previously and strengthens our overall premise of using the basal media for studying bystander effects.

References:

1. Khan S, Shafiei MS, Longoria C, Schoggins JW, Savani RC, Zaki H. 2021. SARS-CoV-2 spike protein induces inflammation via TLR2-dependent activation of the NF-κB pathway. eLife 10:e68563.

2. Ogata AF, Maley AM, Wu C, Gilboa T, Norman M, Lazarovits R, Mao C-P, Newton G, Chang M, Nguyen K, Kamkaew M, Zhu Q, Gibson TE, Ryan ET, Charles RC, Marasco WA, Walt DR. 2020. Ultra-Sensitive Serial Profiling of SARS-CoV-2 Antigens and Antibodies in Plasma to Understand Disease Progression in COVID-19 Patients with Severe Disease. Clinical Chemistry 66:1562–1572.

3. Ruthig VA, Nielsen T, Riel JM, Yamauchi Y, Ortega EA, Salvador Q, Ward MA. 2017. Testicular abnormalities in mice with Y chromosome deficiencies. Biol Reprod 96:694–706.

4. Zheng M, Karki R, Williams EP, Yang D, Fitzpatrick E, Vogel P, Jonsson CB, Kanneganti T-D. 2021. TLR2 senses the SARS-CoV-2 envelope protein to produce inflammatory cytokines. 7. Nat Immunol 22:829–838.

5. Xia B, Shen X, He Y, Pan X, Liu F-L, Wang Y, Yang F, Fang S, Wu Y, Duan Z, Zuo X, Xie Z, Jiang X, Xu L, Chi H, Li S, Meng Q, Zhou H, Zhou Y, Cheng X, Xin X, Jin L, Zhang H-L, Yu D-D, Li M-H, Feng X-L, Chen J, Jiang H, Xiao G, Zheng Y-T, Zhang L-K, Shen J, Li J, Gao Z. 2021. SARS-CoV-2 envelope protein causes acute respiratory distress syndrome (ARDS)-like pathological damages and constitutes an antiviral target. 8. Cell Res 31:847–860.

6. Wu H, Shi L, Wang Q, Cheng L, Zhao X, Chen Q, Jiang Q, Feng M, Li Q, Han D. 2016. Mumps virus-induced innate immune responses in mouse Sertoli and Leydig cells. Sci Rep 6:19507.

7. Wu H, Jiang X et. al. 2019. Mumps virus infection disrupts blood-testis barrier through the induction of TNF-α in Sertoli cells FASEB J 33:12540. doi: 10.1096/fj.201901089R.

8. Pacheco-Hernández LM, Ramírez-Noyola JA, Gómez-García IA, Ignacio-Cortés S, Zúñiga J, Choreño-Parra JA. 2022. Comparing the Cytokine Storms of COVID-19 and Pandemic Influenza. J Interferon Cytokine Res 42:369–392.

9. Zheng M, Karki R, Williams EP, Yang D, Fitzpatrick E, Vogel P, Jonsson CB, Kanneganti T-D. 2021. TLR2 senses the SARS-CoV-2 envelope protein to produce inflammatory cytokines. 7. Nat Immunol 22:829–838.

10. Kumari P, Rothan HA, Natekar JP, Stone S, Pathak H, Strate PG, Arora K, Brinton MA, Kumar M. 2021. Neuroinvasion and Encephalitis Following Intranasal Inoculation of SARS-CoV-2 in K18-hACE2 Mice. 1. Viruses 13:132.

11. Seehusen F, Clark JJ, Sharma P, Bentley EG, Kirby A, Subramaniam K, Wunderlin-Giuliani S, Hughes GL, Patterson EI, Michael BD, Owen A, Hiscox JA, Stewart JP, Kipar A. 2022. Neuroinvasion and Neurotropism by SARS-CoV-2 Variants in the K18-hACE2 Mouse. Viruses 14:1020.

12. Xu J, Qi L, Chi X, Yang J, Wei X, Gong E, Peh S, Gu J. 2006. Orchitis: A Complication of Severe Acute Respiratory Syndrome (SARS)1. Biology of Reproduction 74:410–416.
